# Phospholipase C-ζ-induced Ca^2+^ oscillations cause coincident cytoplasmic movements in human oocytes that failed to fertilize after intracytoplasmic sperm injection

**DOI:** 10.1016/j.fertnstert.2011.12.013

**Published:** 2012-03

**Authors:** Karl Swann, Shane Windsor, Karen Campbell, Khalil Elgmati, Michail Nomikos, Magdalena Zernicka-Goetz, Nazar Amso, F. Anthony Lai, Adrian Thomas, Christopher Graham

**Affiliations:** a School of Medicine, Cardiff University, Heath Park, Cardiff, United Kingdom; b Department of Zoology, University of Oxford, South Parks Road, Oxford, United Kingdom; c IVF Wales, University Hospital of Wales, Heath Park, Cardiff, United Kingdom; d Wellcome Trust/Cancer UK Gurdon Research Institute, University of Cambridge, Tennis Court Road, Cambridge, United Kingdom

**Keywords:** Oocyte, zygote, calcium, movement, phospholipase C zeta, cross correlation, particle image velocimetry

## Abstract

**Objective:**

To evaluate the imaging of cytoplasmic movements in human oocytes as a potential method to monitor the pattern of Ca^2+^ oscillations during activation.

**Design:**

Test of a laboratory technique.

**Setting:**

University medical school research laboratory.

**Patient(s):**

Donated unfertilized human oocytes from intracytoplasmic sperm injection (ICSI) cycles.

**Intervention(s):**

Microinjection of oocytes with phospholipase C (PLC) zeta (ζ) cRNA and a Ca^2+^-sensitive fluorescent dye.

**Main Outcome Measure(s):**

Simultaneous detection of oocyte cytoplasmic movements using particle image velocimetry (PIV) and of Ca^2+^ oscillations using a Ca^2+^-sensitive fluorescent dye.

**Result(s):**

Microinjection of PLCζ cRNA into human oocytes that had failed to fertilize after ICSI resulted in the appearance of prolonged Ca^2+^ oscillations. Each transient Ca^2+^ concentration change was accompanied by a small coordinated movement of the cytoplasm that could be detected using PIV analysis.

**Conclusion(s):**

The occurrence and frequency of cytoplasmic Ca^2+^ oscillations, a critical parameter in activating human zygotes, can be monitored by PIV analysis of cytoplasmic movements. This simple method provides a novel, noninvasive approach to determine in real time the occurrence and frequency of Ca^2+^ oscillations in human zygotes.

It is now firmly established that the cause of oocyte activation at fertilization in animals involves an increase in the cytoplasmic free Ca^2+^ concentration (1). In mammals, the sperm stimulates a prolonged series of Ca^2+^ oscillations, and these have been shown to be essential for oocyte activation and embryo development in mice (1–3). The Ca^2+^ oscillations lead to the stimulation of multiple protein kinases including CaMKII, which is critical for exit from meiotic arrest and entry into the first embryonic cell cycle (2, 4). Distinctive Ca^2+^ oscillations have also been reported in human oocytes after IVF or after intracytoplasmic sperm injection (ICSI) (5, 6). In rabbits and mice, there is also evidence that the precise pattern of Ca^2+^ oscillations during oocyte activation can exert an influence over later developmental parameters, such as implantation rate and the size of the embryo after implantation (4, 7). Recent evidence suggests that the Ca^2+^ oscillations at fertilization are triggered after the sperm fuses with the oocyte, and this leads to the introduction of a sperm-specific phospholipase C-ζ (PLCζ) into the oocyte (8, 9). PLCζ then generates repeated cycles of InsP_3_ production within the ooplasm that cause the repetitive Ca^2+^ release phenomenon of Ca^2+^ oscillations (8, 9). Sperm PLCζ is most conveniently introduced into oocytes by microinjection of its complementary RNA (cRNA), which is translated into PLCζ protein within the oocytes over a period of several hours (10). Hence, the injection of PLCζ cRNA into oocytes produces the PLCζ protein that causes prolonged Ca^2+^ oscillations, and in parallel experiments PLCζ has been shown to activate embryonic development of mouse, pig, cow, and human oocytes up to the morula and blastocyst stages (10–13).

The Ca^2+^ oscillations in mammalian oocytes are routinely detected in research experiments using Ca^2+^-sensitive fluorescent dyes that are loaded into the ooplasm and then excited at specific wavelengths. The combined application of fluorescent dyes and high-intensity excitation light is invasive and, when used at frequencies needed to assess Ca^2+^ oscillations, can cause damage to the oocyte, which then would interfere with later embryo development (14). Hence, the monitoring of Ca^2+^ oscillations with dyes cannot be readily used in combination with longer term studies of embryo development. The invasive and predicted harmful effect of loading oocytes with fluorescent dyes also excludes their use in monitoring the success, or progress, of oocyte activation in any clinical IVF or ICSI treatments. However, it was previously reported that the first few Ca^2+^ transients during fertilization in mouse oocytes are also simultaneously accompanied by abrupt, diminutive movements in the ooplasm (15). We have recently reported that these cytoplasmic movements can be detected over more prolonged periods in mouse zygotes using particle image velocimetry (PIV) analysis (16). In mouse zygotes, this method can be used as a stand-alone technique to monitor the occurrence of Ca^2+^ oscillations in the absence of fluorescent dyes (16). However, as yet, the cytoplasmic movements reported in mouse zygotes have not been described in any other mammalian species. Here we report the use of PIV imaging in human oocytes that were injected with sperm PLCζ cRNA. We demonstrate that sudden but small cytoplasmic movements can be detected in near synchrony with each PLCζ-induced Ca^2+^ transient in human oocytes that had failed to fertilize after ICSI. The method we describe may have potential as a non-invasive method for monitoring Ca^2+^ oscillation patterns in human oocytes.

## Materials and methods

### Human Oocytes

Human oocytes were donated by patients in the IVF Wales clinic at the University Hospital of Wales, Cardiff. The current project and all associated procedures have been approved by the South East Wales Research Ethics Committee and the Human Fertilisation and Embryology Authority (R0161 held by K.S. and N.A.). Aged “failed ICSI” oocytes and fresh oocytes derived from follicle reduction procedures were used. ICSI was performed under standard conditions, and the oocytes were cultured for a further 16–18 hours before they were judged to be fertilized or not. Fresh oocytes were derived from follicle reduction and were used within 5 hours of collection. Only those oocytes that were found to be not activated were used in experiments. During the subsequent 1–4 hours, these oocytes were transferred from the clinic to the research laboratory where they were microinjected with ∼0–20 pL of human PLCζ cRNA, as described elsewhere (10, 11). Briefly, the cRNA was mixed with Ca^2+^-sensitive dye, 0.5 mM Oregon Green BAPTA dextran, (OGBD, InVitrogen). This mixture was then microinjected using a micropipette (∼1-μM tip diameter). The micropipette was inserted into the oocyte using a brief pulse of electrical oscillation from an amplifier that was connected in line to the micropipette needle. A pressure pulse (∼1 second of 20 psi) was then applied to the back of the micropipette to push a bolus of the injection mixture into the oocyte. PLCζ cRNA was prepared as described elsewhere (10). During the data collection, the oocytes were maintained at 37°C in a drop of media under oil, using a Series 40 Quick Change imaging chamber with a CL-100 temperature controller (Warner Instruments). Oocytes were microinjected in M2 media (Sigma), and then recording was in HEPES-buffered KSOM media, as described elsewhere (17).

### Imaging System

The oocytes or zygotes were imaged for several hours after microinjection using a Nikon TiU epifluroescence microscope with a ×20 0.75 NA objective. Fluorescence excitation from a halogen lamp was passed through a 490-nm bandpass filter, reflected via a 505-nm dichroic, and collected with a 530-nm bandpass filter. White light from another halogen lamp was used to visualize the oocytes with differential interference phase contrast (DIC) microscopy. Shutters were placed in the path of these light sources, and filter wheels were in the path of both the fluorescence excitation and emission light such that oocytes were only briefly exposed to light during image acquisition. The shutters and filter wheels were controlled by a Lambda-10 controller (Sutter Instruments). Images were taken with 100- to 200-ms exposures in immediate succession every 10 seconds with a Coolsnap HQ_2_ CCD camera (Photometrics). The Lambda-10, image collection, and initial analysis were controlled using InVivo software (Media Cybernetics) and images stored as tiff stacks.

The fluorescence images were analyzed using ImageJ (http://rsbweb.nih.gov/ij/), and the fluorescence intensity of the dye from the whole oocyte was plotted against time. The movements in the cytoplasm were analyzed with cross-correlation methods that had been developed for studying similar movements in mouse zygotes (16). The algorithm is based on that used in PIV analysis in fluid dynamics research and involves cross-correlating image subregions between successive pairs of images. This analysis gives a vector field representing the movement of local regions of cytoplasm. The mean speed of movement was calculated by taking the mean of the magnitude of the vectors in a square region in the center of the oocyte, as described elsewhere (16). The software was developed and written in MATLAB and is available under an academic, non-commercial use licence at http://users.ox.ac.uk/∼zool0847/code.html.

## Results

**Figure 1:**
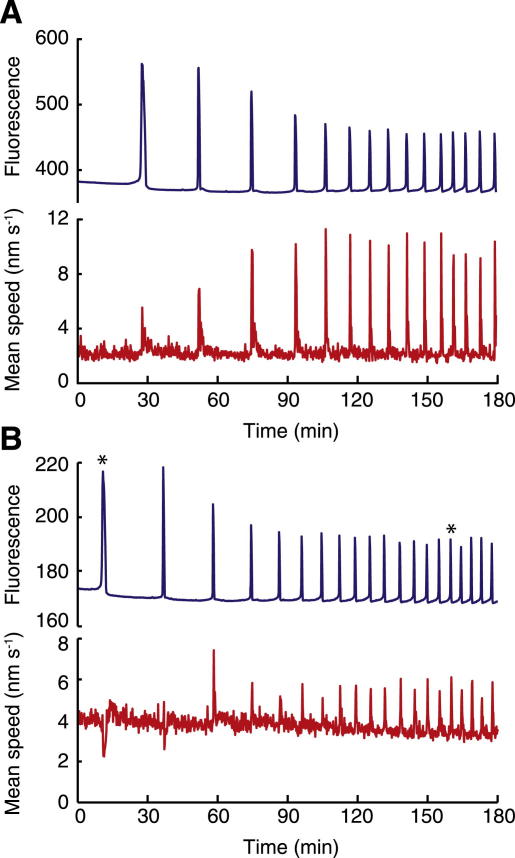
PLCζ- and ICSI-induced Ca^2+^ oscillations in human oocytes are accompanied by coincident transient movements in the oocyte cytoplasm. (**A**) Recording of intracellular Ca^2+^ increases as measured by fluorescence of OGBD (arbitrary units) and the corresponding movements in the cytoplasm as measured with PIV. The traces in (**B**) illustrate the initial phases of Ca^2+^ and PIV, respectively, from another oocyte that showed a large initial Ca^2+^ increase with a decrease in movement. Spikes marked with an asterisk (∗) are shown at an expanded scale in Figure 2.

Microinjection of PLCζ cRNA into human oocytes that had failed to activate after ICSI caused a sustained series of Ca^2+^ oscillations that are illustrated in Figure 1A. The specific pattern of spikes of elevated free Ca^2+^ showed some variation between oocytes, but the general response consisted of a large initial Ca^2+^ increase, followed by a series of smaller Ca^2+^ transients that gradually increased in frequency with time. This general pattern of Ca^2+^ oscillations is similar to that reported elsewhere in human oocytes that were injected with PLCζ cRNA (11). The relatively long latency between cRNA injection (15–20 minutes before the start of recording) and the first appearance of Ca^2+^ spikes and the subsequent build-up in Ca^2+^ spike frequency probably reflect the gradual increase in expression of PLCζ protein with time, which has previously been demonstrated empirically with luciferase-tagged fusion constructs of PLCζ (10, 18–20).

**Figure 2:**
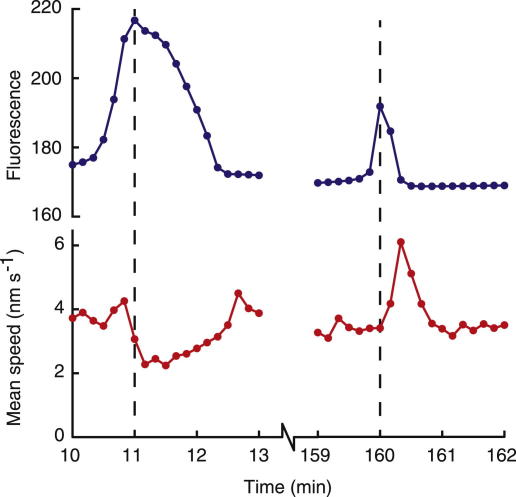
Coincidence analysis of Ca^2+^ changes and cytoplasmic movement. These panels with an expanded time scale are taken from the oocyte in Figure 1B. The first Ca^2+^ spike lasts for a long period, and cytoplasmic movement is suppressed. Later Ca^2+^ spikes are shorter, and the mean magnitude of movement in the PIV speed peak increases to a maximum shortly after the spike maximum. In the illustrated case, the speed-peak maximum occurs 20 seconds after the Ca^2+^ maximum. Note that the mean magnitude of movement starts to accelerate within the same 10-second interval as the Ca^2+^ maximum.

When we analyzed the PLCζ cRNA-injected oocytes for cytoplasmic movements using PIV, we found that distinct movements occurred within the same 10-second interval, or just after each Ca^2+^ transient (Fig. 1B). In these human oocytes, the maximum mean PIV speed was either coincident with the maximum of the Ca^2+^ spike or it occurred within the 50 seconds after the maximum of the Ca^2+^ transient. In total, 95/102 cytoplasmic movements were detected within this range from 10 different zygotes. The mean lag of the PIV speed peak was 18 seconds after the Ca^2+^ peak (with a range of 10–50 seconds). It is noteworthy that the movements detected in the oocyte cytoplasm are relatively small in scale: the mean magnitude of all vectors at a speed peak never exceeded 40 nm/s, and within these speed peaks, local regions never moved faster than 120 nm/s. Even though in most cases there was a sudden increase in movement associated with a Ca^2+^ transient, it was also notable that the higher level of Ca^2+^ achieved in the first spike of some recordings was often accompanied by reduced movement in the cytoplasm (four of six cases; Fig. 1B). In one case, when the Ca^2+^ level remained high at the second peak, the cytoplasmic movement was also suppressed (one of six; Figs. 1B and 2).

**Figure 3:**
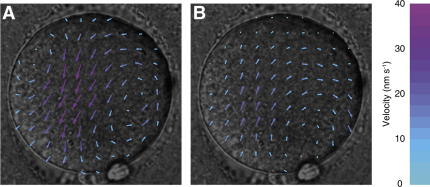
Images of a human oocyte overlaid with the pattern of cytoplasmic movement. Each vector map records movement during the 10-second interval between one frame and the next. (A and B) This pair of vector maps were collected 20 seconds apart, and they illustrate the reversal of movement direction without alteration in movement orientation.

For individual oocytes, the vectors of movement had a consistent orientation during each speed peak, but the direction of movement was often reversed within a spasm of movement (Fig. 3). However, the mean direction of movement had no consistent relationship with the position of the first or second polar bodies. In addition, small vortices were frequently visible in speed peaks. Given the limitation of the small number of oocytes imaged in a single plane, these vortices were not obviously associated with any particular oocyte structure.

The degree of synchrony between Ca^2+^ elevation and movement was the common finding in all of these human oocytes. This suggests that the cytoplasmic movements in human oocytes are directly induced by elevated free Ca^2+^ ions. Previous studies in mouse zygotes showed that such movements in the cytoplasm are dependent on the actin cytoskeleton and are greatly influenced by the presence of the sperm and fertilization cone (16). It was difficult to identify the region of the activated human oocytes that changed shape during the speed peaks, although there were slow progressive changes of cell profile detected during the recordings. In some records it was possible to observe a granular region moving around the cytoplasm and forming a thick granular crescent beneath the cell membrane. This peripheral crescent also moved in relation to the position of the first polar body, but the precise crescent boundary was poorly defined. It remains unclear whether any potential sperm may be present within these failed ICSI oocytes or if any structures around a sperm could play a specific role in cytoplasmic movements, as suggested in mouse zygotes. However, we did carry out a similar analysis of PLCζ cRNA-induced Ca^2+^ oscillations and cytoplasmic movements in seven unfertilized oocytes that were obtained after follicle reduction. Notably, we failed to detect any movements associated with the 41 Ca^2+^ spikes observed in these follicle reduction–derived oocytes.

## Discussion

In this study we have shown that Ca^2+^ transients induced by PLCζ in human oocytes that failed to fertilize after ICSI are accompanied by transient cytoplasmic movements, or spasms, that can be detected using PIV analysis of oocyte DIC images collected using a CCD camera. Previous studies have reported movements in the cytoplasm of human zygotes during the 1-cell stage, but these have a slow cycle time (∼30 minutes) and are not related directly to activation events (21). Our study is the first to report repetitive cytoplasmic movements in human oocytes occurring within a brief period of tens of seconds. Moreover, these movements are clearly correlated with the precise timing of Ca^2+^ increases that occur repetitively in response to injection of sperm PLCζ cRNA.

Previous work in mouse zygotes has demonstrated the role of myosin light chain kinase and the actin cytoskeleton in promoting the cytoplasmic movements (16). In these studies, the presence and location of a sperm and the fertilization cone was important. In this study, it is notable that we observed the cytoplasmic movements only in failed ICSI oocytes but not in unfertilized human oocytes that were obtained from follicle reduction where Ca^2+^ oscillations were also induced by the injection of PLCζ. This could be explained by the greater postovulatory age of the oocytes that had failed to fertilize after ICSI. Alternatively, although we did not verify sperm incorporation in our oocytes, the potential presence of a sperm after ICSI could also be important for effective observation of cytoplasmic movements in human oocytes.

The repetitive spikes in Ca^2+^ concentration previously described in human oocytes after IVF or ICSI occur about once every 10–30 minutes (5, 6). This is similar to the range of Ca^2+^ spike frequencies that we observed in the initial period after injection in our previous and current experiments involving microinjecting PLCζ cRNA (11). The available evidence points to the basic amplitude and shape of Ca^2+^ increases that are triggered by PLCζ being identical to those seen at fertilization in mouse, bovine, and pig oocytes (9). Consequently, the pattern of Ca^2+^ oscillations that we have induced with PLCζ injection should be a close mimic of the corresponding pattern detected in normal fertilization.

It remains unclear at present whether a single parameter, such as the total number of Ca^2+^ spikes, will prove to be that which predicts the success rate of development of the zygote to the blastocyst stage in humans. In the mouse and rabbit, the number of Ca^2+^ spikes can influence the timing and degree of oocyte activation and later development (4, 7). There may be more factors involved in human zygote development since the rate of development to the blastocyst in humans is reduced compared with the mouse strains commonly used. Very few studies have addressed the role of Ca^2+^ oscillations in human embryo development. In possibly the only relevant study, oocytes that failed to fertilize after ICSI were subjected to one or three electroporation pulses to generate one or three Ca^2+^ transients (22). Both groups of oocytes were successfully activated and cleaved to the 2-cell stage at similar rates, but those oocytes exposed to three Ca^2+^ pulses showed greater development to the blastocyst stage than those receiving one single Ca^2+^ pulse. This type of study has not been followed up, in part because of the technical constraints imposed by the requirement to measure Ca^2+^ in the same oocyte that is then assessed for embryo development. Our present approach of monitoring cytoplasmic movements with PIV offers a potential method that could be used in future for studies of such oocytes to relate specific changes (e.g., timing, frequency, amplitude) in the cytoplasmic Ca^2+^ profile to the success rate of human preimplantation embryo development.

A recent study found that frequent imaging of the developing human embryo can be used to predict the ability of the zygote to develop to the blastocyst (23). Correlations have been made, for example, with regards to the timing of pronuclear formation, the time taken for the initial rounds of mitosis, and later embryonic development to the blastocyst stage (23). Some of these factors have been related to the timing of Ca^2+^ oscillations in mouse oocytes (24). The studies of early mouse, hamster, and human embryos have established some of the limits of light tolerance of developing zygotes. It is evident that human embryos can develop normally, to blastocyst or even to full term, when exposed to light for <1 second every few minutes for periods of several days (25, 26). To use our method of monitoring cytoplasmic movements as an indicator of successful fertilization or development potential, it will probably be necessary to expose human zygotes to light every 10 seconds for several hours. However, it remains to be determined whether this level of light exposure will have any detrimental effect on the rate of embryo development.

The data in this study show that the temporal analysis of oocyte cytoplasmic movements can provide a directly correlative measure of the number and timing of Ca^2+^ transients occurring in aged human oocytes that failed to fertilize after ICSI. This has the potential to be a simple and minimally invasive clinical method for assessing the occurrence of Ca^2+^ oscillations in such oocytes. Studies of mouse fertilization have suggested that the analysis of such cytoplasmic movements (i.e., to report the simultaneous Ca^2+^ transients) may provide an early and effective indication of zygote viability after IVF (16). Future studies will have to confirm that such movements also occur during IVF and ICSI of non-aged human oocytes and that the light exposure during the requisite imaging is not harmful to embryo development.

## References

[1] Stricker S.A. (1999). Comparative biology of calcium signaling during fertilization and egg activation in animals. Dev Biol.

[2] Ducibella T., Fissore R. (2008). The roles of Ca^2+^, downstream protein kinases signaling in regulating fertilization and the activation of development. Dev Biol.

[3] Kline D., Kline J.T. (1992). Repetitive calcium transients and the role of calcium in exocytosis and cell cycle activation in the mouse egg. Dev Biol.

[4] Ducibella T., Schultz R.M., Ozil J.P. (2006). Role of calcium signals in early development. Semin Cell Dev Biol.

[5] Taylor C.T., Lawrence Y.M., Kingsland C.R., Biljan M.M., Cuthbertson K.S.R. (1993). Oscillations in intracellular free calcium induced by spermatozoa in human oocytes at fertilization. Hum Reprod.

[6] Tesarik J., Sousa M., Testart J. (1994). Human oocyte activation after intracytoplasmic sperm injection. Hum Reprod.

[7] Ozil J.P. (1998). Role of calcium oscillations in mammalian egg activation: experimental approach. Biophys Chem.

[8] Saunders C.M., Larman M.G., Parrington J., Cox L.J., Royse J., Blayney L.M. (2002). PLCζ: a sperm-specific trigger of Ca^2+^ oscillations in eggs and embryo development. Development.

[9] Swann K., Saunders C.M., Rogers N., Lai F.A. (2006). PLCζ(zeta): a sperm protein that triggers Ca^2+^ oscillations and egg activation in mammals. Sem Cell Dev Biol.

[10] Yu Y., Saunders C.M., Lai F.A., Swann K. (2008). Preimplantation development of mouse oocytes activated by different levels of human phospholipase Czeta. Hum Reprod.

[11] Rogers N.T., Hobson E., Pickering S., Lai F.A., Braude P., Swann K. (2004). PLCζ causes Ca^2+^ oscillations and parthenogenetic activation of human oocytes. Reproduction.

[12] Yoneda A., Kashima M., Yoshida S., Terada K., Nakagwa S., Sakamoto A. (2006). Molecular cloning, testicular expression and oocyte activation potential of porcine phospholipase C zeta. Reproduction.

[13] Ross P.J., Beyhan Z., Iager A.E., Yoon S.Y., Schellander K., Fissore R.A. (2008). Parthenogenetic activation of bovine oocytes using bovine and murine phospholipase C zeta. BMC Dev Biol.

[14] Squirrell J.M., Wokosin D.L., White J.G., Bavister B.D. (1999). Long-term two-photon fluorescence imaging of mammalian embryos without compromising viability. Nat Biotechnol.

[15] Deguchi R., Shirakawa H., Oda S., Mohri T., Miyazaki S. (2000). Spatiotemporal analysis of Ca^2+^ waves in relation to sperm entry site and animal-vegetal axis during Ca^2+^ oscillations in fertilized mouse eggs. Dev Biol.

[16] Ajduk A., Ilozue T., Windsor S., Yu Y., Seres K.B., Bomphrey R.J. (2011). Rhythmic actomyosin-driven contractions induced by sperm entry predict mammalian embryo viability. Nat Commun.

[17] Summers M.C., Bhatnagar P.R., Lawitts J.A., Biggers J.D. (1995). Fertilization in vitro of mouse ova from inbred and outbred strains: complete preimplantation embryo development in glucose-supplemented KSOM. Biol Reprod.

[18] Nomikos M., Blayney L.M., Larman M.G., Campbell K., Rossbach A., Saunders C.M. (2005). Role of phospholipase C-ζ domains in Ca2+-dependent phosphatidylinositol 4,5-bisphosphate hydrolysis and cytoplasmic Ca2+ oscillations. J Biol Chem.

[19] Nomikos M., Elgmati K., Theodoridou M., Calver B.L., Cumbes B., Nounesis G. (2011). Male infertility-linked point mutation disrupts the Ca2+ oscillation-inducing and PIP(2) hydrolysis activity of sperm PLCzeta. Biochem J.

[20] Nomikos M., Elgmati K., Theodoridou M., Calver B.L., Nounesis G., Swann K. (2011). Phospholipase Cζ binding to PtdIns(4,5)P_2_ requires the XY-linker region. J Cell Sci.

[21] Payne D., Flaherty S.P., Barry M.F., Matthews C.D. (1997). Preliminary observations on polar body extrusion and pronuclear formation in human oocytes using time-lapse video cinematography. Hum Reprod.

[22] Zhang J., Wang C.W., Blaszcysk A., Grifo J.A., Ozil J.P., Haberman B.A. (1999). Electrical activation and in vitro development of human oocytes that fail to fertilize after intracytoplasmic sperm injection. Fertil Steril.

[23] Wong C.C., Loewke K.E., Bossert N.L., Behr B., De Jonge C.J., Baer T.M. (2010). Non-invasive imaging of human embryos before embryonic genome activation predicts development to the blastocyst stage. Nat Biotechnol.

[24] Lawrence Y., Ozil J.P., Swann K. (1998). The effects of a Ca^2+^ chelator and heavy metal ion chelator upon Ca^2+^ oscillations and activation at fertilization in mouse eggs suggest a role for repetitive Ca^2+^ increases. Biochem J.

[25] Pribenszky C., Mátyás S., Kovács P., Losonczi E., Zádori J., Vajta G. (2010). Pregnancy achieved by transfer of a single blastocyst selected by time-lapse monitoring. Reprod Biomed Online.

[26] Nakahara T., Iwase A., Goto M., Harata T., Suzuki M., Ienaga M. (2010). Evaluation of the safety of time-lapse observations for human embryos. J Assist Reprod Genet.

